# Visual–Motor Functions and Associated Cognitive Outcomes in Pediatric Cancer Survivors

**DOI:** 10.3390/medsci13020041

**Published:** 2025-04-05

**Authors:** Alena Deviaterikova

**Affiliations:** Research Institute for Brain Development and Peak Performance, RUDN University, Mikluho-Maklaya, Str., 11, Moscow 117198, Russia; alena.deviaterikova@gmail.com

**Keywords:** visual–motor ability, childhood cancer, late effects, executive functions

## Abstract

Introduction: Pediatric cancer survivors are at high risk for visual–motor and cognitive deficits that persist throughout life. These domains are related to academic performance. The current study examined (i) whether both visuomotor and cognitive functions and (ii) whether visuomotor functions alone mediate the relationship between age and cognitive functions. Methods: In total, there were 210 participants (7–17 years): 70 posterior fossa tumors (M_age_ = 12.1  ±  3.2 years, 44% female) and 70 acute lymphoblastic leukemia (M_age_ = 12.3  ±  3.4 years, 45% female) survivors and 70 (M_age_ = 12.2 ± 3.3 years, 41% female) healthy controls. Visual motor integration, motor coordination and visual perception were assessed using the Beery VMI test. Working memory, attention and planning were assessed using CANTAB. Results: Impaired motor function is significantly more pronounced than cognitive impairment in both groups of cancer survivors (effect size from 25 to 30% for visual–motor and from 5 to 7% for cognitive functions). A multiple regression model revealed that age and visual motor functions are significant predictors of attention (in the ALL group β = −0.490, t = −4.88, *p* = 0.000) and working memory (in the PFT group β = 0.264, t = 2.72, *p* = 0.008; in the ALL group β = 0.215, t = 2.24, *p* = 0.028). Conclusions: In children who have experienced acute lymphoblastic leukemia and tumors of the posterior cranial fossa, visual–motor dysfunction is more pronounced than cognitive impairment. In addition, there is an association between visual–motor function disorders and working memory. These findings can be used to develop more specific rehabilitation protocols.

## 1. Introduction

Pediatric cancer survivors have a high risk for a variety of late effects, with patients frequently reporting impaired cognitive and motor functions. Visual–motor and cognitive are known determinants of academic performance. Children who have survived cancer demonstrate academic performance that is below that of their healthy peers [1]. However, it remains unclear to what extent motor and cognitive functions are impaired in children and how these disorders are related.

The most common cancers among children are posterior fossa tumors (PFTs) of the cerebellum and acute lymphoblastic leukemia (ALL). ALL is a malignancy that originates in lymphoid cells in the bone marrow and can spread throughout the body and central nervous system. Treatment protocols for these oncological diseases suggest that children experience varying degrees of neurotoxicity, which manifests as a decrease in cognitive and motor function [2]. In PFT protocols, the tumor itself is usually removed in the first stage, and then additional treatment methods are used—chemotherapy or radiation therapy—that are also aimed at the central nervous system [3]. Thus, in both types of pediatric cancer, chemotherapy is aimed at the head and neck area. Such treatment results in a decrease in the white matter of the brain, thereby disrupting the conduction of nerve impulses [4]. A decrease in the conductivity of the white matter of the brain is common, leading to a subsequent decrease in visual–motor and cognitive functions [5,6].

The survival rate of children has now reached 70–95% for PFT and ALL, and with the help of modern treatment methods with surgery and chemotherapy, it is approaching its limits. Despite the high survival rates, the treatment remains highly toxic and causes late effects. Recent tractography studies have shown white matter damage as a biomarker of treatment-related neurotoxicity in PFTs survivors [7]. The loss of white matter leads to a decrease in cognitive and motor functions, and results in academic achievement decrease [8,9,10]. Around 40% children report cognitive and motor impairments [11]. In the absence of additional rehabilitation, these impairments are likely to persist throughout life and to reduce its quality [12].

It is now evident that childhood cancer leads to a decrease in cognitive functions [13]. Individual studies [14,15] and systematic reviews have shown that the cognitive functions of children who have survived cancer can be improved through rehabilitation [16,17].

However, most rehabilitation methods are often aimed specifically at cognitive rehabilitation [18]. That is, when cognitive functions decline, children receive various forms of cognitive training. At the same time, there is evidence that interventions aimed at training motor skills are also effective in improving cognitive functions [14,19].

Nevertheless, it is not yet completely clear which disorders are primary and which functions should be prioritized as a rehabilitation target to increase the rehabilitation effect.

All previous studies have had small and disparate samples of children with various diagnoses and treatment protocols. Therefore, we selected two groups of children with the most common malignant oncologic diseases: PTF and ALL, treated according to the same protocols. Each group included 70 children who had completed treatment at least three months prior to the testing and achieved remission status. We aimed to evaluate the deficit of cognitive and visual–motor functions in children who survived cancer compared to healthy peers and to determine whether these disorders are related.

The identification of similar cognitive and visual–motor deficits in different oncologic diseases affecting the central nervous system will allow for the development of universal rather than personalized rehabilitation programs, as well as the elaboration of systemic rehabilitation programs that are aimed at comprehensive recovery rather than restoring a specific function.

## 2. Materials and Methods

### 2.1. Participants

A total of 210 children participated in this study. A convenience sample of 70 posterior fossa tumors of cerebellum (M_age_ = 12.1  ±  3.2 years, 44% female) and 70 acute lymphoblastic leukemia (M_age_ = 12.3  ±  3.4 years, 45% female) survivors, and 70 healthy controls (M_age_ = 12.2 ± 3.3 years, 41% female) was assessed. Children with PFT received treatment according to the HIT MED 2017 protocol, and children who survived ALL received treatment according to the ALL—Moscow—Berlin 2008 and ALL—Moscow—Berlin 2015 protocols. In both cases, the children had malignant tumors and were receiving treatment through high-dose chemotherapy in the head and neck area. At the time of testing, all the children had completed cancer treatment and were in remission after first diagnosis.

The remission period in both cancer groups did not differ, ranging from 24 to 72 months, with the majority of children attaining remission status at the age of 7.5 years (that is, the older the child, the more time has passed since the disease). The testing procedure was a one-time operation, conducted by qualified neuropsychologists. Testing took about an hour with a 15 min break between cognitive and visual–motor tests. A medical psychologist assessed the patients at the Clinical Rehabilitation Research Center “Russkoye Pole” at the Dmitry Rogachev National Medical Research Center of Pediatric Hematology, Oncology and Immunology. The control group was tested by a school psychologist.

Inclusion criteria: age over 7, remission of the disease, IQ above 80 on the Raven test, and for patients with malignant neoplasm of the posterior cranial fossa of the cerebellum or acute lymphoblastic leukemia, chemotherapy for the head and neck area. Exclusion criteria: amaurosis, deafness, severe motor deficits, significant visual impairment, visual field impairment, partial optic nerve atrophy.

This study was approved by the Ethics Committee of Dmitry Rogachev National Medical Research Center of Pediatric Hematology, Oncology and Immunology before the training (protocol number 8e/13–17 of 27 October 2017). The study protocol was developed in accordance with the Declaration of Helsinki. All participants, or their parents/legal representatives/guardians, gave informed consent after receiving detailed information about this study.

### 2.2. Measures

The following background variables were assessed: age and biological sex, recorded from questionnaires; age at diagnosis, cancer type, treatment duration, and type of treatment were obtained from medical records.

The Cambridge Neuropsychological Test Automated Battery (CANTAB) is a validated computerized test battery used to measure cognitive functions [20]. This battery provides a rapid and interactive assessment of a child’s cognitive functioning. At the same time, all tasks are non-verbal and performed using a touchpad. We evaluated the functions that are associated with success in school—attention, working memory and planning [21].

This study used three tests to assess working memory (SSP—spatial span), attention (RVP—Rapid Visual Information Processing), and planning function (SOC—Stockings of Cambridge).

In SSP, the screen displays white squares, some of which briefly change color in different sequences. The participant must memorize which squares lit up. Then, they must select the squares that changed color in the same order in which they were displayed by the computer. The number of squares in the sequence increases from two at the beginning of the test to nine at the end, and the order and color change during the test. In our study, we recorded the maximum length of the sequence that the child could keep in memory.

In the RVP test, a white field is displayed in the center of the screen in which numbers from 2 to 9 appear in a pseudo-random order at a rate of 100 digits per minute. The participants are asked to identify the target sequence of numbers (3-5-7). When the participant sees the target sequence, they must respond by pressing the button in the center of the screen as quickly as possible. We considered the number of errors (false clicks) made by the participants.

In the SSP test, a participant is shown two images. In each of these images, three stockings with three colored balls are suspended from a beam. Two images are displayed at the top and bottom of the screen. The balls are positioned differently in each image. The participant must move the balls in the image at the bottom of the screen to copy the pattern shown in the image at the top. The balls are moved one at a time by first selecting the desired ball and then by selecting the position to which it is to be moved. The participant is asked to make as few moves as possible to match the two patterns. In this test, the recorded parameter was the number of tasks completed in the minimum number of moves.

The Beery–Buktenica Developmental Test of Visual–Motor Integration—(Beery VMI test) is a standardized test to assess visual perceptual skills and visual motor functions in children [22]. The test consists of three subtests. In the first subtest, the participant must copy the shapes according to the pattern (visual–motor integration). In the second part, the participant must find a shape that is the same as a sample of similar shapes (visual perception). And in the third subtest, the participant must connect the lines inside the shape without touching the edges of the shape (motor coordination). Each subtest evaluates the correctness of the task and provides a standardized score. In our study, we compared participants by standardized scores.

#### Common Terminology Criteria for AEs (CTCAE)

An integrative indicator of the CTCAE v 4.0 toxicity scale was used to assess the “toxicity of the treatment” [23]. The integrative indicator includes the following scales: hematological toxicity, blood loss, infection, blood clotting disorder (blood counts, the presence of blood loss, the presence and severity of infection, blood clotting disorder); gastrointestinal toxicity (assessment of somatic disorders such as anorexia, diarrhea, dry mouth, heartburn, etc.); cardiovascular toxicity (disorders of the cardiovascular system, such as arrhythmia, blood pressure, heart attack, etc.); liver toxicity (disruption of liver systems, such as alkaline phosphatase, bilirubin, liver enlargement, etc.); pulmonary toxicity (disruption of the respiratory system, such as pulmonary edema, hiccups, shortness of breath, etc.); genitourinary system (for example, cystitis, urinary incontinence, etc.); neurological toxicity (for example, hearing loss, headache, etc.); flu-like syndrome, allergies, skin changes; metabolic and endocrine disorders; ocular and dental changes; and bone-related injuries. This questionnaire is filled out by the attending physician, according to a 5-point system where 0—no toxicity, 1—mild toxicity, 2—moderate toxicity, 3—severe toxicity and 4—life-threatening toxicity.

### 2.3. Statistical Analysis

The statistical analysis was conducted using the Jamovi statistical package (Ver. 2.6.24.) [24].

First, we assessed whether there were differences between the three groups of children using a one-factor analysis of variance, as well as the size of the effect of these differences.

Second, we assessed whether there was an influence of gender, age, and the presence of cancer on the indicators of visual–motor and cognitive functions.

Finally, using regression analysis, we assessed which factors made a significant contribution to the indicators of working memory, planning and attention in three groups of children.

## 3. Results

Using a one-factor analysis of variance, we analyzed whether there was a difference in the three groups of children (Table 1).

Children who survived PFT showed significantly more pronounced cognitive and motor deficits compared to children who survived ALL and the control group. Children who experienced ALL and PFT demonstrated the most pronounced motor deficits (effect size from 25 to 30% in visual–motor functions, and from 4 to 7% in cognitive functions).

We assessed the contribution of confounding variables, such as age, sex and oncology. A multifactorial analysis of variance was performed to evaluate the factors. As a result, oncology (*p* = 0.000) and age (*p* = 0.000) became significant factors influencing the indicators of cognitive and visual–motor functions. The results of the analysis did not reveal interfacial interaction (*p* < 0.05), i.e., the factors influence in isolation (Table 2).

Since the factors were independent, the next step was to perform a multidimensional regression analysis to determine the main contribution of the independent factors (sex, age, and oncology) to the cognitive and motor functions of the children.

As a result of multiple regression in the PFT group, three predictors explained 38% of the variance in the working memory index—age, treatment toxicity, and motor coordination. In the group of children after ALL, three factors were also identified—age, visual perception, and visual–motor integration, which explained 39% of the variance in working memory. In the control group, the model included only one significant factor, age, which accounted for 18% of the variance in working memory (Table 3).

As a result of multiple regression, a significant factor that contributed to the model in the group with PFT was age, which accounted for 4% of the variance in attention. In the group after ALL, visual–motor integration and age emerged as significant factors, explaining 26% of the variance in attention indicators. In the control group, two significant factors were also identified—gender and age, which together explained 13% of the error variance in the attention task (Table 4).

Sex, accounting for 6% of the variance, was a significant predictor influencing the planning function in the group of children who survived PFT. In the group of ALL children, age was found to be an important predictor, explaining 13% of the variance in the indicators of the planning function. In the control group, two significant factors contributing to the planning function were identified—age and visual–motor integration, explaining 13% of the variance in the planning indicators (Table 5).

## 4. Discussion

In this study, we investigated the presence of (i) visual–motor and cognitive deficits in pediatric cancer survivors, and (ii) the severity of this deficiency, and determined whether these were disorders. First, the results showed that visual–motor and cognitive functions were reduced compared to the control group. The important result was that the decrease in visual–motor functions was more pronounced than the decrease in cognitive functions.

A more pronounced decrease in visual–motor functions, compared with cognitive ones, is important for the preparation of subsequent rehabilitation protocols. Most protocols are aimed at targeted training of cognitive functions, while visual–motor functions also need rehabilitation. We assumed that visual–motor impairments affect cognitive ones, thus representing a complex disorder.

However, in our study, we identified the contribution of visual–motor functions only to indicators of working memory, and no such relationship was found with planning and attention. Perhaps this is due to the close connection between working memory and the cerebellum, especially in the group of children who survived tumors of the posterior fossa.

The age factor played a significant role in all groups of children—in PFT, ALL and healthy controls (*p* = 0.000). This is an important finding that shows that the development of cognitive and visual–motor functions continues despite the cancer. In general, even without special rehabilitation, children who have survived cancer can adapt to society with varying degrees of success [25]. Older children who survived cancer in early childhood show worse results than the control group. This confirms the need for targeted rehabilitation in children who have survived cancer.

Despite the fact that oncological diseases are less common in girls than in boys, some studies show that the effect of the disease is more harmful in boys than in girls [26]. However, in our study, gender did not significantly contribute to the indicators of cognitive and visual–motor functions of children. Perhaps the sample was insufficient for the analysis, or the participation of children with more pronounced cognitive and visual–motor difficulties is necessary in order for the effect of gender to be more pronounced.

The results are a step toward explaining how motor and cognitive impairments after cancer can be related and mutually determined. In our study, we found that visual–motor functions contribute to working memory performance in both cancer groups.

Research on new motor learning shows that working memory makes a significant contribution to learning a new visual–motor skill [27]. By now, we can definitely say that visual–motor functions and working memory are closely related, and this relationship is confirmed by a common brain substrate, the cerebellum. Neuroimaging studies show that the cerebellum is activated both when solving the n-back task (a traditional task to assess working memory capacity) and when solving visual–motor tasks [28].

At the same time, there was no relationship between visual–motor functions and the functions of attention and planning [29]. But, even in these studies, the relationship between working memory and visual–motor functions was most pronounced [30].

The close relationship between working memory and visual–motor functions suggests that they are interrelated. For practice, this means that rehabilitation should not only aim at restoring cognitive functions but should be comprehensive—this way, we can achieve a greater effect and improve the quality of life of patients [31,32].

This study has several limitations, including a small sample size and the inability to apply complex statistical analysis methods. Another limitation is the lack of data on the socioeconomic status of the participants, which could have contributed to the research conclusions. One more limitation is that we do not know what level of cognitive and visual–motor function development the children had prior to diagnosis and treatment. In addition, we were able to obtain data on their school performance, which means that we cannot draw conclusions about how the cognitive and visual–motor deficits identified affect the quality of life of children. We are therefore certain that the results of this study are valuable for research (e.g., design of interventions) and clinical practice (e.g., assessment of deficits and rehabilitation).

## 5. Conclusions

This study showed the peculiarities of the development of cognitive and visual–motor functions in children who survived acute lymphoblastic leukemia and tumors of the posterior cranial fossa. Both cancer groups showed a significant decrease in function compared to the control group. The data obtained on the relationship between visual–motor functions and working memory can serve as a basis for the development of more effective training programs that are based not only on cognitive training. Future research should focus on a more complex analysis of the structure of the multidirectional relationship between working memory and visual–motor functions and other cognitive functions. These studies will help to develop more effective rehabilitation programs.

## Figures and Tables

**Table 1:** The results of a one-factor analysis of variance in cognitive and visual–motor functions in three groups of children.

	ALL	PFT	Control	F	*p*	Effect Size
	Mean (SD)	Mean (SD)	Mean (SD)			
SSP (sequence length)	5.2 (1.6)	4.7 (1.8)	6.2 (1.4)	18.6	0.000	7%
Attention (mistakes)	3.7 (5.2)	5.8 (8.7)	3.7 (2.9)	6.8	0.012	4%
Planning (correctly solved tasks)	6.9 (2.2)	6.3 (2.2)	7.5 (2.2)	7.5	0.000	5%
VMI (visual–motor integration)	98.4 (9.6)	90.2 (14.5)	108.4 (8.5)	65.1	0.000	30%
VP (visual perception)	101.8 (12.3)	88.4 (14.1)	104.2 (9.2)	49.6	0.000	25%
MC (motor coordination)	94.7 (12.8)	86.2 (15.7)	105.4 (10.2)	53.6	0.000	26%

Note: SD—standard deviation, F—Fisher’s criterion, *p*—significance level.

**Table 2:** Assessment of the impact of oncology, sex and age on cognitive and motor functions.

	F	Effect df	Error df	*p*
Oncology	4.440	30	352.0	0.000
Sex	1.304	15	176.0	0.203
Age	5.778	30	352.0	0.000

Note: *p*—significance level.

**Table 3:** The results of the regression analysis for working memory for three groups of school-aged children.

Group	R^2^	Corr. R^2^	Predictor	β	B	T	F	*p*
ALL	0.647	0.394	Age	0.633	0.311 (0.048)	6.44	16.79	0.000
			Visual perception	0.251	0.031 (0.013)	2.48		0.015
			Visual motor integration	0.215	0.037 (0.017)	2.24		0.028
PFT	0.641	0.388	Age	0.617	0.047 (0.312)	6.59	17.47	0.000
			Toxicity	−0.276	0.036 (−0.099)	−2.78		0.007
			Visual motor integration	0.264	0.012 (0.033)	2.72		0.008
Control	0.440	0.186	Age	0.440	0.046 (0.224)	4.83	23.35	0.000

Note: R^2^ is the coefficient of determination, Corr. R^2^—coefficient of determination, β—regression coefficient, B—standard error, t—Student’s criterion, F—Fisher’s criterion, *p*—significance level.

**Table 4:** The results of the regression analysis for attention for three groups of school-aged children.

Group	R^2^	Corr. R^2^	Predictor	β	B	T	F	*p*
ALL	0.535	0.267	visual motor integration	−0.490	−0.165 (0.034)	−4.88	14.26	0.000
			age	−0.235	−0.230 (0.098)	2.34		0.001
PFT	0.240	0.045	age	−0.240	0.298 (−0.643)	−2.15	4.65	0.034
Control	0.391	0.135	age	−0.335	0.171 (−0.611)	−3.57	8.65	0.000
			sex	0.203	0.990 (2.135)	2.15		0.001

Note: R^2^ is the coefficient of determination, Corr. R^2^—coefficient of determination, β—regression coefficient, B—standard error, t—Student’s criterion, F—Fisher’s criterion, *p*—significance level.

**Table 5:** The results of the regression analysis for planning for three groups of school-aged children.

Group	R^2^	Corr. R^2^	Predictor	β	B	T	F	*p*
ALL	0.379	0.131	age	−0.294	0.055 (−0.170)	−3.10	8.44	0.003
PFT	0.461	0.064	sex	0.278	0.506 (1.424)	2.37	8.91	0.000
Control	0.387	0.132	age	−0.294	0.55 (−0.170)	−3.10	8.44	0.003
			sex	−0.289	0.019 (−0.057)	−3.04		0.003

Note: R^2^ is the coefficient of determination, Corr. R^2^—coefficient of determination, β—regression coefficient, B—standard error, t—Student’s criterion, F—Fisher’s criterion, *p*—significance level.

## Data Availability

The datasets generated during and/or analyzed during the current study are available from the corresponding author on reasonable request.

## Abbreviations

The following abbreviations are used in this manuscript:

PFT	Posterior Fossa Tumors
ALL	Acute Lymphoblastic Leukemia
CANTAB	Cambridge Neuropsychological Test Automated Battery
VMI	Visual–Motor Integration
VP	Visual Perception
MC	Motor Coordination
